# Rare occurrence of small bowel intussusception due to synchronous metastasis of renal cell carcinoma

**DOI:** 10.1590/0102-67202025000036e1905

**Published:** 2025-10-27

**Authors:** Matheus Felipe Ferreira AGUIAR, Rodrigo Ambar PINTO, Ulysses RIBEIRO-JUNIOR, Pedro Castro SOARES, Carlos Frederico Sparapan MARQUES

**Affiliations:** 1 Universidade de São Paulo, Faculty of Medicine, Cancer Institute of Sao Paulo State, Gastrintestinal Unit – São Paulo (SP), Brazil.; 2 Universidade de São Paulo, Faculty of Medicine, Cancer Institute of Sao Paulo State, Pathology Unit – São Paulo (SP), Brazil.

**Keywords:** Intussusception, Carcinoma, renal cell, Intestinal obstruction, Intussuscepção, Carcinoma de células renais, Obstrução intestinal

## Abstract

**Background:**

Renal carcinoma is the third most common urological cancer, with 30% of patients presenting with metastases at diagnosis. Metastases to the small intestine are rare (0.7–1.1%), and their presentation as intestinal intussusception is even more uncommon, with only a few cases reported in the literature.

**Aims:**

The aim of the study was to present a case of stage IV clear cell renal carcinoma with a rare presentation of intestinal intussusception, leading to emergency department admission due to severe anemia and melena.

**Methods:**

A 62-year-old man presented with melena for 2 months and a critically low hemoglobin level of 2.9 g/dL (normal range: 13.5–17.5 g/dL). Abdominal and pelvic angiotomography identified an exophytic lesion in the left kidney consistent with renal carcinoma and an approximately 16 cm ileal intussusception.

**Results:**

Exploratory laparotomy revealed intestinal intussusception and a 4 cm lesion on the antimesenteric border, suspected to be a tumor. A segmental resection with primary anastomosis was performed, resulting in a favorable postoperative recovery. Histopathological and immunohistochemical analyses confirmed poorly differentiated metastatic clear cell renal carcinoma.

**Conclusions:**

This report underscores the need to consider gastrointestinal symptoms in patients with renal carcinoma, as an intestinal metastasis, although rare, is a potential complication. Synchronous metastases are even rarer and present a significant diagnostic challenge.

## ARTICLE HIGHLIGHTS

Small bowel metastasis from renal cell carcinoma is extremely rare and can present as intussusception, with few cases reported in the literature.

A 62-year-old male presented with anemia and melena and was diagnosed with synchronous small bowel metastasis causing ileal intussusception.

Surgical resection of the affected bowel segment resulted in symptom resolution and allowed initiation of systemic therapy.

This case underscores the importance of considering intestinal metastasis in renal cancer patients with gastrointestinal bleeding or obstructive symptoms.

## Summary

**CENTRAL MESSAGE:**

Renal carcinoma is the third most common urological cancer and the seventh among all neoplasms. Approximately 30% of patients with renal carcinoma present with metastases at diagnosis, with the most frequent sites being the lungs (50–60%), bones (30–40%), liver (3040%), and brain (5%). Metastases to the small bowel are extremely rare (0.7–1.1%) and are usually found when there is diffuse dissemination of the primary tumor, typically presenting as iron deficiency anemia due to occult bleeding or obstructive symptoms.

**PERSPECTIVES:**

The presented report underscores the importance of maintaining suspicion for symptoms such as gastrointestinal bleeding, anemia, and intestinal obstruction in patients with renal carcinoma, considering the possibility, albeit rare, of intestinal metastasis. It is important to note that in cases of synchronous metastases, the diagnosis is even more uncommon, and the level of suspicion is more challenging.

## VISUAL ABSTRACT

**Figure:**
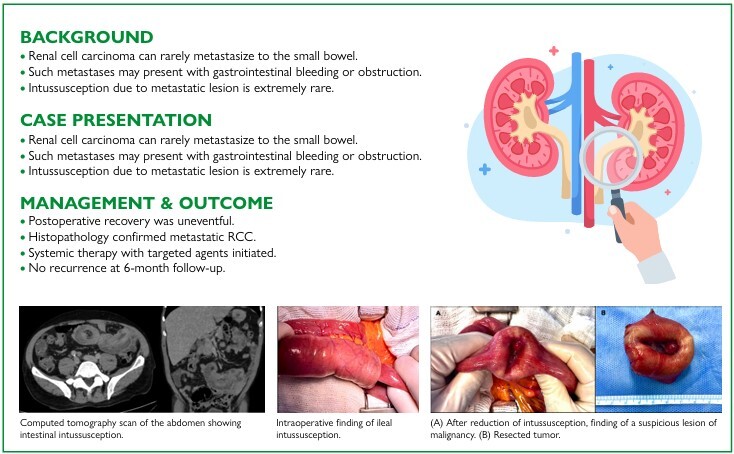

## RESUMO

**Racional:**

O carcinoma renal é o terceiro câncer urológico mais frequente, sendo que 30% dos pacientes apresentam metástases já ao diagnóstico. Raramente são encontradas metástases no intestino delgado (0,7–1,1%), e ainda menos frequente é a apresentação da metástase como intussuscepção intestinal, com poucos relatos na literatura até então.

**Objetivos:**

Relatar o caso de paciente com carcinoma de células claras renais em estágio IV, cuja rara apresentação foi manifestada por intussuscepção intestinal e admissão em serviço de emergência por anemia grave e melena.

**Métodos:**

Homem, 62 anos, admitido com melena há 2 meses e achado crítico de hemoglobina de 2,9 g/dL (normal 13,5–17,5g/dL). Realizada angiotomografia de abdome e pelve com achado de lesão exofítica em rim esquerdo compatível com neoplasia primária e intussuscepção de alça ileal, por cerca de 16 cm.

**Resultados:**

Foi submetido a laparotomia exploradora, com identificação de intussuscepção intestinal a 80 cm do ângulo de Treitz, e lesão de 4 cm na borda antimesentérica, suspeita para acometimento neoplásico. Foi realizada enterectomia segmentar com anastomose primária, com boa evolução pós operatória. O resultado anatomopatológico e imunohistoquímica foram compatíveis com carcinoma pouco diferenciado células claras renais metastático.

**Conclusões:**

O doente apresentado traz a importância de se manter suspeição para sintomas gastrointestinais em pacientes com diagnóstico de carcinoma renal, tendo em vista a possibilidade, ainda que em raros casos, de metástase intestinal. Há de se ressaltar, que em caso de metástases sincrônicas, o diagnóstico é ainda mais incomum e o grau de suspeição mais desafiador.

## INTRODUCTION

 Renal carcinoma is the third most common urological cancer and the seventh among all neoplasms, with an estimated annual incidence of around 430,000 cases and 179,000 deaths worldwide^16^ . The most common subtype is clear cell renal carcinoma (RCC), which accounts for 70–80% of cases^6,13,15^. 

 Approximately 30% of patients with RCC present with metastases at diagnosis, with the most frequent sites being the lungs (50–60%), bones (30–40%), liver (30–40%), and brain (5%)^4,8,20^. Metastases to the small bowel are extremely rare (0.7–1.1%) and are usually found when there is diffuse dissemination of the primary tumor, typically presenting as iron deficiency anemia due to occult bleeding or obstructive symptoms. Even more rarely, RCC metastasis can present as intestinal intussusception, with only a few reports in the literature to date^3^ . 

 Intestinal intussusception is more common in adults, representing 1–5% of cases of intestinal obstruction. Unlike the pediatric population, in adults, it is usually secondary to some underlying pathology, with two-thirds of cases caused by neoplasms, 50% of which are malignant. Treatment typically involves surgical intervention with resection of the affected intestinal segment^2,10,19,20^ . 

 The objective is to report the case of a patient with stage IV clear cell RCC, metastatic to bones, lymph nodes, and small bowel. This rare synchronous presentation was manifested by intestinal intussusception, leading to admission to the emergency department for severe anemia and melena. 

## METHODS

 A 62-year-old black male presented to the emergency department with symptoms of weakness, hypotension, pallor, and abdominal pain. His recent history included right shoulder pain for approximately 5 months and melena for 2 months, with no etiological investigation conducted to date. He had no significant personal medical history and denied comorbidities, medication use, smoking, or illicit drug use. Family history was negative for neoplasms or hereditary diseases. On physical examination, the patient had a fair general condition, was pale, and had a slightly distended and tender abdomen with a palpable mass in the mid-abdomen. Rectal examination revealed melena. 

 Initially, the possibility of upper gastrointestinal bleeding was considered. Clinical monitoring and stabilization of the patient were initiated, along with laboratory tests, which revealed a critical hemoglobin level of 2.9 g/dL (normal range 13.5–17.5 g/dL). 

**Figure 1:**
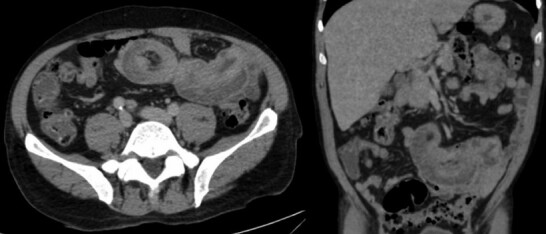
Computed tomography scan of the abdomen showing intestinal intussusception.

**Figure 2:**
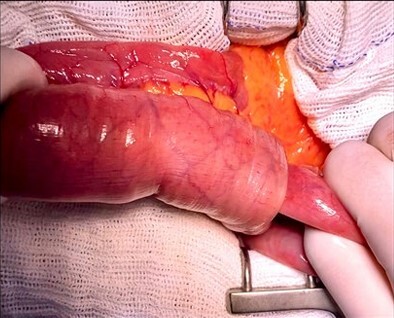
Intraoperative finding of ileal intussusception.

**Figure 3:**
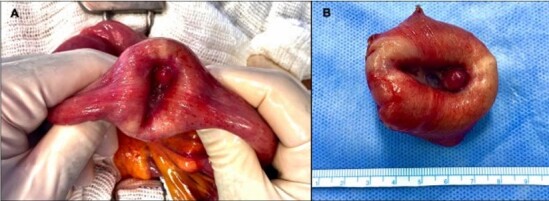
**(A)** After reduction of intussusception, finding of a suspicious lesion of malignancy. **(B)** Resected tumor.

 After hemodynamic resuscitation and transfusion support, an upper gastrointestinal endoscopy was performed, which did not show any lesions or signs of active bleeding. Subsequently, a computed tomography (CT) scan of the abdomen and pelvis (Figure 1) was conducted, revealing the following findings: an exophytic lesion in the left kidney consistent with a primary neoplasm; an expanding mass in the right adrenal gland; bilateral pulmonary nodules; mediastinal lymphadenopathy; lytic lesions in the head of the right humerus and pelvis, suspected to be secondary involvement; and intussusception of an ileal loop, approximately 16 cm in length, with thickening of the distal segment of the intussuscepted loop (Figures 2 and 3). 

 With effective hemodynamic and transfusion support and based on the examination findings, surgical intervention was indicated. Intestinal intussusception was considered the likely etiology of chronic gastrointestinal bleeding. An exploratory laparotomy was performed, identifying intussusception of an ileal segment 80 cm from the Treitz angle, extending for 15 cm, without bowel ischemia or necrosis (Figure 2). 

 After the reduction of the intussuscepted segment, a 4-cm lesion was identified on the antimesenteric border, suspected to be neoplastic (Figure 3A). A segmental resection with a lateral-to-lateral anastomosis using a mechanical stapler was promptly performed (Figure 3B). 

 The procedure was completed without complications, and the patient was transferred to the ward, showing good postoperative progress. An oral diet was initiated on the 2nd postoperative day (POD), and the patient was discharged from the hospital on the 5th POD. 

 Outpatient follow-up and first-line systemic chemotherapy with Pazopanib 800 mg once daily were started. The histopathological results revealed poorly differentiated carcinoma with a small cell pattern infiltrating the segment of the small intestine up to the subserosa, along with angiolymphatic invasion and clear margins. 

**Figure 4:**
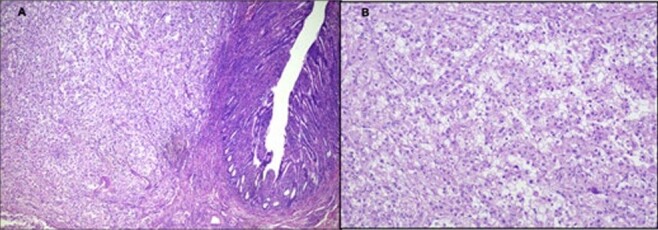
Pathological finding of the surgical specimen. **(A)** The results of the histologic examination were consistent with metastatic renal clear cell carcinoma (magnification 40×). **(B)** Hematoxylin and eosin (H&E), 100× magnification showing small bowel resection specimen.

**Figure 5:**
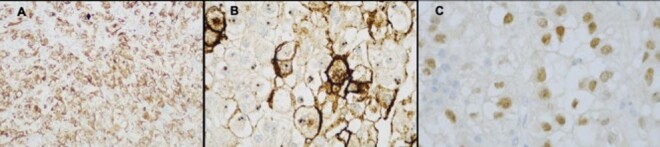
Immunohistochemical analysis of the tumor revealing: **(A)** AEI/AEI3 (100×), an epithelial cell marker confirming carcinoma. **(B)** CD10 (400×), a marker that stains clear cell carcinoma. **(C)** PAX-8 (400×), a marker indicating a renal origin.

 Immunohistochemistry confirmed the diagnosis of intestinal metastasis from RCC, with positive results for CD10 (Common Acute Lymphoblastic Leukemia Antigen), PAX8 (Paired-box gene 8), and AE1/AE3 (Cytokeratins), findings consistent with metastatic clear cell RCC (Figures 4 and 5). 

**Figure 6:**
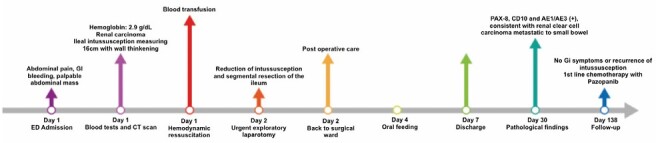
Case timeline.

 After 6 months of follow-up, the patient had not experienced any new episodes of bleeding, obstructive symptoms, or the development of new metastatic lesions in the small intestine. Figure 6 demonstrates the patient’s evolution from hospital admission until the last follow-up visit. The patient signed the Informed Consent form for this publication. 

## DISCUSSION

 Clear cell RCC has high metastatic potential, with onethird of patients presenting with metastases at the time of the primary diagnosis. However, the main point of interest in the presented case is the rare occurrence of metastasis to the small bowel, given that the more common sites are the lungs, lymph nodes, and bones^4,5^. 

 The rarity of this case underscores the importance of documenting such instances to better understand their presentation patterns and clinical implications, particularly since it presented as intestinal intussusception, an even rarer event. 

 Diagnosing this condition is challenging due to the variable and non-specific symptoms, which commonly include abdominal pain, nausea, vomiting, gastrointestinal bleeding, anemia, and, more rarely, intussusception. This variability can lead to misdiagnosis or delayed treatment. For synchronous metastases in patients without other symptoms suggesting renal tumors, as in the presented case, the diagnosis and level of suspicion become even more challenging^9^. 

 A study of 3,637 patients with RCC identified 26 cases (0.71%) of gastrointestinal metastasis. Gastrointestinal bleeding was the most common clinical manifestation, present in 67% of the cases^12^. A literature review found only 46 cases of metastasis to the small bowel reported since 2000^11^. 

 Another study^14^ involving 21 patients with gastrointestinal metastases from RCC found 8 (38%) with intestinal intussusception. These patients were predominantly men with a mean age of 62 years, with the most common location being the jejunum, and the interval between the diagnosis of the primary tumor and the metastasis ranged from 5 to 6 years. The present case had the same age range, but ileal involvement of the bowel metastasis in parallel to the primary RCC. 

 A recent literature review^7^ identified 99 published cases from 1950 to 2022, with only 10% being synchronous metastases. Of these cases, 83% were men, with a mean age of 63 years. The most frequent symptoms were gastrointestinal bleeding and abdominal pain, and the most commonly used diagnostic method was CT. Most patients underwent metastasectomy, and the 5-year survival rate was 36%. Another recent literature review^17^ identified 60 cases of metastatic RCC to the small intestine, with five cases presenting as intussusception. 

 A frequent explanation for the delayed diagnosis of intestinal metastases is the location of the lesions. Even in patients with gastrointestinal bleeding, endoscopy and colonoscopy may fail to identify such lesions due to challenging access and location in some cases^14^. 

 In our case, the diagnosis was made through the identification of significant ileal intussusception on a contrast-enhanced abdominal CT scan (Figure 2), which has an accuracy ranging from 58 to 100%. Classic findings include "target," "bulls-eye," or sausage-shaped lesions, mesenteric vessels within the bowel lumen, and air in the bowel wall due to necrosis or gangrene^10^. 

 Surgical treatment is generally the preferred approach for intestinal intussusception in adults. When the etiology is metastatic RCC, the treatment remains controversial, taking into account the patient’s clinical condition, number, and resectability of the lesions. However, when opting for surgery, it is important to consider not only the potential for symptom palliation but also improvement in overall survival^1,18^. 

## CONCLUSIONS

 The presented report underscores the importance of maintaining suspicion for symptoms such as gastrointestinal bleeding, anemia, and intestinal obstruction in patients with RCC, considering the possibility, albeit rare, of intestinal metastasis. It is important to note that in cases of synchronous metastases, the diagnosis is even more uncommon, and the level of suspicion is more challenging. 

 Furthermore, two aspects warrant consideration: first, the diagnosis, which is based on the unique presentation of symptoms in each patient, as demonstrated in the case presented, where CT identified the intestinal intussusception. Second, the choice of treatment, which, based on clinical conditions and resectability, should always consider the potential opportunity for metastasectomy with radical resection of the intestinal lesion, aiming for symptomatic control and improved survival. 

## Data Availability

The information regarding the investigation, methodology, and data analysis of the article is archived under the responsibility of the authors.
